# Service user perspectives on engagement in an occupational therapy-led pulmonary rehabilitation programme: A qualitative interview study

**DOI:** 10.1177/03080226221103155

**Published:** 2022-06-02

**Authors:** Gemma Bradley, Leigh Rooney, Phillip J Whitehead

**Affiliations:** 1 Department of Social Work, Education and Community Wellbeing, 5995 Northumbria University, Newcastle Upon Tyne, UK; 2 Population Health Sciences Institute, 12186 Newcastle University, Newcastle Upon Tyne, UK

**Keywords:** Pulmonary rehabilitation, service user experiences, doing, being, becoming, belonging

## Abstract

**Introduction:**

Pulmonary rehabilitation (PR) is an intervention for people with chronic respiratory conditions. There are questions about which components are important to its success, including the nature of occupational therapy involvement. The aim of this research was to explore the experiences of people who had attended an occupational therapy-led PR programme in the United Kingdom to determine the most important components.

**Method:**

Semi-structured telephone interviews were conducted with service users who had experience of a community-based PR programme. Interviews were transcribed verbatim. Data were analysed using the framework analysis method with three researchers contributing to the analysis.

**Findings:**

Nine people took part in the interviews, with a mean age of 72 years. Four themes were identified which were organised around the concepts of Doing, Being, Becoming Belonging. These were ‘Doing exercise and physical activity’, ‘being breathless’, ‘belonging as an individual within the group’ and ‘becoming a person who lives with Chronic Obstructive Pulmonary Disease’.

**Conclusion:**

Doing physical activity, whilst coping with being breathless and belonging as an individual within a group can positively influence experiences and perceived outcomes during and after PR. These dimensions have the potential to shape occupation-focussed PR programmes and the occupational therapy contribution in this area of practice.

## Introduction

There are an estimated 1.2 million people living with Chronic Obstructive Pulmonary Disease (COPD) in the UK with 115,000 people newly diagnosed each year (British Lung Foundation, 2021). The main progressive symptoms of COPD include dyspnea (shortness of breath), chronic cough and sputum production (Williams et al., 2011). These symptoms, alongside the fact that COPD commonly co-exists with other conditions such as coronary heart disease, heart failure, malnutrition, sleep disorders, anxiety and depression mean that the impact on daily life is significant. There is currently no cure for COPD and therefore services are focused on diagnosis and management (World Health Organisation, 2021).

Pulmonary Rehabilitation (PR) is established as an essential management strategy for COPD and is defined as a comprehensive and tailored intervention that includes exercise training, education and strategies to promote behaviour change. It is designed to improve the physical and psychological condition of people with chronic respiratory disease and to promote the long-term adherence to health-enhancing behaviours (Spruit et al., 2013).

PR is usually a multi-disciplinary intervention with supervised exercise training being an integral part. Beyond this, there is limited consensus about the specific components essential to effectiveness, such as which professionals should be involved, what the exercise training should include and how long it should last (McCarthy et al., 2015; Troosters et al., 2019). Research which further investigates the most important components for service users is therefore essential.

Perhaps because the exercise component is so central, involvement of physiotherapists, respiratory nurses and exercise-training specialists is commonly cited (Troosters et al., 2019). Occupational therapy offers potential to contribute to, and lead, PR however research exploring involvement has been limited. This qualitative study explores the service user experience of engaging in an occupational therapy-led PR programme and seeks to explore the components of this programme important in its effectiveness from user perspectives.

## Literature

Evidence suggests that PR leads to improvements in health-related quality of life, dyspnea, fatigue, emotional wellbeing and exercise capacity (McCarthy et al., 2015). Exercise is recommended as a central feature of PR (National Institute of Health and Care Excellence, 2018) with many benefits for people with COPD, and the co-morbidities that commonly co-exist with chronic respiratory disease. However, a recent review highlighted benefits of exercise-based interventions cannot be assumed and that there are many complex and interacting factors which influence both participation and outcomes (Brighton et al., 2020). For example, early evidence suggested outcomes were better for groups who undertook hospital-based programmes (McCarthy et al., 2015) but more recent evidence supports community-based PR and telerehabilitation as safe, feasible and effective (Cecins et al., 2017; Cox et al., 2021).

Alongside studies which synthesise the impact of PR on a range of outcomes, understanding the service user experience of PR is of interest, with issues of adherence and response to programmes being particularly important. Brighton et al. (2020) explored the experiences, needs and preferences of people with COPD and frailty who participated in outpatient PR. They found that participants were highly motivated to participate in PR although their changeable health led to multiple disruptions completing programmes, often leading to missed sessions, non-completion or reducing motivation. Oates et al. (2019) found that factors supporting adherence to a PR programme included personal determination, support from peers, family and friends, and programme features such as motivational staff and engaging education. Belonging to a group as part of a PR programme has also been highlighted as important in adherence, with informality of the atmosphere and group leaders who foster social connectedness identified as positive features to promote engagement (Halding et al., 2010).

The philosophy of occupational therapy – including a focus on motivation, self-determination and the social environment – is not only strongly aligned with the principles of PR, but presents important opportunities to understand challenges with adherence, completion and sustainability of change. There are examples which orientate PR around the central concept of occupation (Easthaugh et al., 2019), yet a review of published studies yielded very few examples which specifically mention occupational therapy for the management of COPD (Walker et al., 2016) and across the limited available studies findings are inconclusive. Vaes et al. (2019) found that a PR programme which supplemented exercise training with an occupational therapy-led energy conservation intervention led to improvement in performance of activities of daily living (ADL). However, without a control group or a design which accounts for the complexity of the intervention, it is difficult to attribute any change to particular elements. Although not specifically PR, a small-scale randomised controlled trial comparing occupational therapy interventions for people with COPD with usual care found no improvement in self-reported occupational performance and satisfaction within the intervention group, but did report that there were significant changes in activity performance (Martinsen et al., 2016).

Further research is needed in order to understand the different components of PR including which professionals are involved and the location of delivery (McCarthy et al., 2015). Furthermore, research which strengthens the evidence base for occupational therapy in the management of COPD, and more specifically the contribution of occupational therapy to PR, is required. This research aimed to contribute to these priorities through exploring service user experiences of an occupational therapy-led PR programme.

## Method

### Design

A qualitative research design was employed using semi-structured interviews to explore individual experiences of people who had undertaken a PR programme within one NHS Trust in northern England. A research advisory group (consisting of clinicians, service users who had participated in previous programmes and the research team) met before study recruitment. The group advised on the content of study information material and the interview schedule.

### Recruitment of participants

Participants starting PR programmes in January 2020 were give a leaflet about the study by the lead occupational therapist, asked for initial expressions of interest and asked for verbal consent to share contact details with the research team. The clinical team determined whether participants met the study inclusion criteria in that people needed to be able to give consent for involvement and able to communicate in English for the purpose of an interview. Sixteen people gave consent to be contacted and were sent a full participant information sheet by post. A researcher then contacted individuals by telephone to give them an opportunity to ask questions about the research.

### The programme

Study participants were involved in a PR programme delivered over 6 weeks, with two 2-hour sessions per week. All sessions were facilitated by a minimum of one lead occupational therapist and an exercise trainer and included an educational component and a supervised exercise component. Some sessions also included multi-disciplinary contributors for different educational topics which included inhaler techniques, diet and nutrition and mental health and wellbeing. Although some topics were arranged in advance (in order to pre-book multi-disciplinary facilitators), others were discussed and prioritised within the group, in conjunction with the lead occupational therapists.

### Data collection

**Figure 1.**
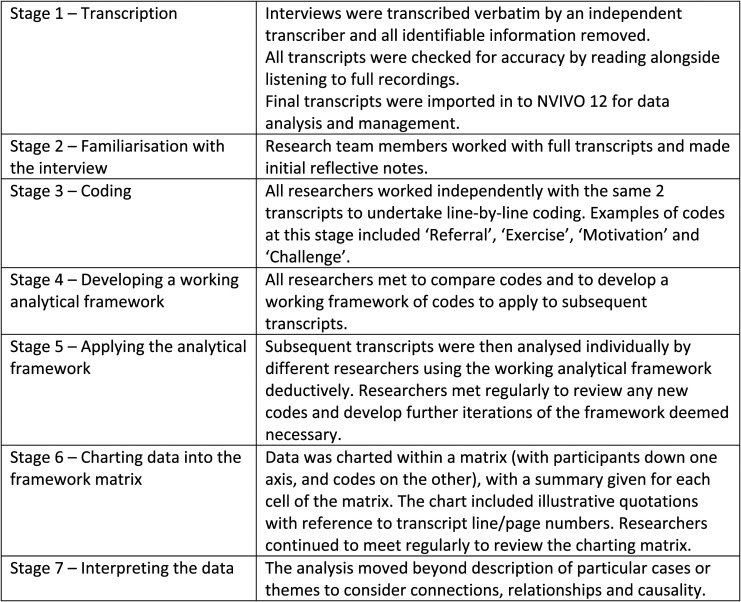
Stages of framework analysis as outlined by Gale et al. (2013).

An interview schedule was developed collaboratively by members of the research team, clinical team and with guidance from the advisory group. The interview schedule covered questions relating to relating to referral to the programme, daily life before and after the programme and strengths and limitations of the programme. A copy of the full interview schedule can be obtained from the corresponding author. Due to the research taking place during the first phase of the COVID-19 pandemic, face-to-face interviews were changed to telephone interviews. All interviews were conducted by the same member of the research team (LR) and were audio-recorded and transcribed verbatim with all identifiable information omitted from the transcript. Interview data were analysed by all authors using framework analysis, an approach particularly useful when multiple researchers are working with data (Gale et al., 2013). This method (outlined in Figure 1) enables the organisation of data by both case (individual participants) and by code, allowing themes to be established across cases whilst also retaining individual participant accounts.

### Ethical considerations

Ethical approval was obtained from the Health Research Authority and from the Higher Education Institution of the corresponding author in November 2019. Permissions were also granted from the NHS Trust Research and Development Department. Researchers involved in data collection and analysis were independent of the clinical team who delivered the PR programme.

## Findings

From 16 people who were sent study information, five declined to be involved after receiving the information and two people did not respond to the follow-up contact. Therefore, nine participants took part in the study, six female and three male, and with a mean age of 72. Two lived alone and seven with a spouse. All were retired. Telephone interviews lasted between 31 min and 61 min.

The PR programme was delivered across three different leisure centres and all venues were represented in the sample. Three participants attended all 12 sessions, with others missing between one or four sessions. Although the reasons varied, many of those missing sessions reported this was due to their own or family members’ ill health.

### Overview of themes

Wilcock’s Occupational Perspective of Health (2001a, 2001b, 2006) presents four dimensions of occupation – doing, being, belonging, becoming – and suggests that these dimensions are essential to health. In our analysis, these four concepts and the relationships between them were identified as being central to participants’ experiences of the PR programme and we have therefore used this framework to illustrate how engagement in PR can become a meaningful occupation itself, alongside being a vehicle to enable engagement in wider occupations, both of which are essential to health. These four dimensions are captured in the first extract below:

Just actually being able to go out and even go to the shop. I didn’t feel I could go on my own because I think I had been so poorly and short of breath. I use a walking stick anyway. I just felt vulnerable being outside, so doing this group was the start of the journey, you know, the journey that I’ve done with them and what I’ve learnt and what I’ve been able to overcome with the group (Participant 5).

Participant 5 highlights elements of each of the concepts which form the structure for our four themes. The importance of ‘*doing* this group’ points to our first theme of ‘Doing exercise and physical activity’. Having ‘been so poorly and out of breath’ reflects the second theme of ‘Being breathless’ as a fundamental aspect of living with COPD. ‘The journey that I’ve done with them and […] what I’ve been able to overcome with the group’ emphasises the social connections developed within the programme, the importance of *belonging* to the group and to life *becoming* something different after the programme – our third and fourth themes.

### Doing exercise and physical activity

And we started doing these exercises and I found I could manage them, and I wasn’t having any problems. I mean, so using muscles that I hadn’t used for a long, long time, but then as the weeks went on, I was finding that I was able to do them like longer and faster than when I had started. So, it was definitely an improvement on my physical health as well (Participant 6).

In the extract above, Participant 6 described the beneficial effect of ‘doing these exercises’ and the impact this had on their bodies. This was coupled with ability to exercise for increased durations and at increased intensities – ‘I was able to do them like longer and faster’ – as the programme progressed. This was related to overall improvements outside of the programme – ‘on my physical health as well’. Participants linked ‘doing exercise and physical activity’ to beneficial effects both within and outside of the group, including immediately at the time of the doing.

As soon as we started exercising and things like that, I loved it and I chatted to maybe the girl who was next to me, or the man who was next to me (Participant 3).

Participant 3 highlights how being engaged in doing exercises helped her to talk and connect with the other group members, suggesting a link between doing and belonging which is discussed further in a later section. Participants highlighted that ‘doing’ provided specific opportunities for learning, and PR session facilitators were valued for providing their expertise which led to learning while doing

When I was doing my exercises there’s times she would come over and say, you’ve turned a funny colour, stop a bit, you know (Participant 4).

The learning and confidence experienced whilst doing exercise could also translate to confidence for future ‘doing’

just getting the confidence from doing the exercises […] they gave you more confidence to even like walk up the bank, you know. How to control your breathing if you were getting really, really breathless (Participant 6).

It is important to note that not all participants referred to success when doing exercises

It’s not a bad thing, it’s just being able to do it…the majority of the time, I can’t exercise because of my breathing. […] I mean, I just couldn’t exercise and that’s how I thought sometimes the emphasis on exercise…it wasn’t particularly good for some patients (Participant 2).

This extract provides insight into the experience of not being able to ‘do’ the exercises, which led to a negative perception of this element of the programme, illustrated by the reflection that it ‘wasn’t particularly good’ for some members.

### Being breathless

They talked about exercising was good for you, breathing… actually if you got out of breath a little bit, that was good, because it got your heart beating and it got your lungs [working] a bit (Participant 9).

The state of ‘being breathless’ was an important component of the PR programme and built upon the component of ‘doing’. The doing of exercise within the programme enabled Participant 9 to get ‘out of breath a little’ which came to be accepted as something ‘good’. This is in contrast with extracts which refer to understanding of breathlessness prior to the programme, often discussing the state of being breathless as one which was worrying or preoccupying participants.

But I find as though I’m uncomfortable...not stressful, uncomfortable when I am trying to exercise because I know that I am going to be out of breath, and I don’t like to be out of breath to be honest (Participant 2).

Here, Participant 2 describes the state of being breathless as uncomfortable, a state which she does not ‘like’. Although she states it is ‘not stressful’ the inclusion of the word stressful does add to the sense of unpleasantness which she experiences. Some participants went further to suggest that the anticipation of being breathless inhibited them from carrying out activities, with one suggesting they stayed in the house because they were ‘frightened to get out of breath’ (Participant 3). A key aspect of the programme was that it educated, enabled and encouraged participants to engage with the concept of moderate breathlessness.

Probably before it [the programme] I thought, you’ve got COPD and asthma, don’t over-do yourself, you know, but that’s a bit of a fallacy because exercises, they talked about exercising was good for you, breathing… actually if you got out of breath a little bit, that was good (Participant 9).

The reference to ‘before’ the programme suggests a changing understanding on the part of Participant 9 and that a certain degree of breathlessness was appropriate and encouraged during activity. In this extract, we also see the link between the previous theme: doing the exercise within the confines of the programme enabled participants to experience breathlessness in a way which was no longer uncomfortable (as in the previous extract) but was now ‘good’.

As with the previous theme, there were challenges for some participants enacting the concept of being breathless to a moderate degree within the programme.

See I can’t moderate breathe, that’s the thing though, you see. I just was out of breath and then that was it, I was out of breath (Participant 2).

Participant 2 illustrates that being moderately breathless was not something easily achieved. This is the same participant from the previous theme who felt that the emphasis on exercise was not a positive one for all participants, which emphasises the links between doing and being [breathless] and the feedback between them. In this interpretation, the ‘doing’ would lead to an uncomfortable sense of being which leads the participant to stop and not feel engaged in doing.

### Belonging as an individual within the group

There were two aspects to ‘belonging’ identified within the data and we present them separately here. First, questions of ‘Do I belong?’ in this programme were common amongst participants when the referral was first suggested to them and when they joined the group. Second, ‘Belonging as an individual within the group’ captures the importance of social connections to others, but that this was balanced with personalised and tailored (individual) approaches.

### Do I belong?

And when I went for my yearly check up in January this year… She says, ‘Well how did you find the rehabilitation?…I felt awful and I said, ‘I didn’t contact them’. She said, ‘Why not?’ and I explained. I didn’t think it was for me. I didn’t think that I was that poorly that I needed it (Participant 7).

Several participants initially questioned whether they belonged to the group – ‘I didn’t think it was for me’ – either before they started or during the early sessions. Thinking they were not ‘that poorly’ suggests assumptions about who they thought the group should be for, an evaluation that they did not fit with this and subsequently resulted in a decision not to go. Questioning the sense of belonging also presented itself once participants did start the programme. Participants compared themselves and their own situations to others and using this as some form of measure to judge the relevance of being part of the programme.

I think that I was probably the fittest there. I felt a little bit, kind of, of a fraud (Participant 1).

Participants’ sense of belonging was also linked to the physical space. As the programme took place in leisure facilities, it was acknowledged that people living with COPD may be ‘put off’ by feelings of not belonging in that space and therefore efforts were made by facilitators to alleviate such concerns

At the 5th week, we did our normal routine, but they said to us there, we’re actually going to go into the gym, which was next door to the room we were in. [The facilitator said] don’t be put off by it, by the word gym, you know, you’re going to a gym. It’s not like it used to be years ago, it’s not all muscle bound 18–19 year old kids lifting heavy weights or anything (Participant 9).

### Belonging as an individual within the group

So, the whole thing was you thought that…I’m going to be meeting these same people for the next sort of 6 weeks, twice a week, you know, twice a week and literally it just became a very friendly little group with everybody helping one another, you know, if one was sort of stuck on one exercise (Participant 6).

The sense of connection to others as people engaged in activities was an important part of the programme experience. ‘Everybody helping one another’ suggests mutual support, reciprocity and contribution to others were important. An earlier extract from Participant 3 highlighted that connections were formed whilst *doing* the exercises, and this was also highlighted when people referred to the enjoyment or competition that was shared when doing particular exercises. However, social connections to others did not only develop whilst doing activities. The opportunities for reflection and ‘joint discussion’ within the group could potentially provide affirmation to members, and help people to recognise they were ‘better than they thought’:

I could see in some people’s faces and some of the remarks afterwards, discussions. Like a joint discussion, you know like a group discussion that they found that they could actually manage it better than they thought they would (Participant 1).

Once such connections were formed, participants seemed to value belonging to a consistent group of people.

I did like sticking to the same people because you get to know, well not so much the people, she [the facilitator] got to know you and you knew exactly… She knew what you could do in the exercising (Participant 3).

‘[S]ticking to the same people’ suggests that consistency within the group was valued, and reference to the fact that ‘she got to know you’ also shows the importance of the connection to the facilitator. In particular, by reflecting that the facilitator got to know ‘what you could do in the exercising’, this connection was also linked to the facilitator’s understanding of individual abilities in relation to doing.

Despite the importance of individuality within the group, there was also an emphasis on belonging as an individual in this theme. A sense of belonging was not about homogeneity and it was seen as a positive element when individual differences were respected:

the opening group session when she went through everything and said everybody is at different levels but we’ll cater for everybody and it will benefit everybody kind of thing, you know, so I kind of settled my thoughts down (Participant 1).

However, challenges were raised by some people in terms of their sense of belonging, particularly in relation to feeling like an individual within a large group:

But I mean you are talking about 15 people all of different degrees of COPD…they [the facilitators] can’t spread themselves individually when they’ve got 15 in the class (Participant 2).

Again, this extract is from the same participant who experienced challenges with carrying out exercises and achieving a state of moderate breathlessness. Participant 2 also seemed to feel less of a sense of belonging as an individual within the group, in contrast to some other participants. This further supports the links between doing, being and belonging.

### Becoming a person who lives with chronic obstructive pulmonary disease

I’ve got more confidence. More confidence back and I can do more now. I do tend to do a little bit more exercise, which was something that I… because of the arthritis as well, that I wasn’t doing. […] I can go down the stairs and have a little walk up and down the street. (Participant 5).

This account from Participant 5 of becoming a person with ‘more confidence’ and a person who ‘can do more’ was a common narrative from many participants. Another participant made reference to an increase in confidence, a reduction in fear and creating a more positive vision of the future:

Unfortunately my father had emphysema […] and I think I was getting it into my mind that that was the way that I was going to end up, like exactly the same as my father who I mean, well he ended up with… well he died with it…[However, since the programme] I’ve got no fear that I am going to end up like my father, so mentally I just feel more confident about facing the future (Participant 6).

There were many stories of change following the programme with examples of walking further, managing stairs with more ease, taking up the opportunity for gym membership, making dietary changes or changing inhaler regimes. When viewed as a whole, people made links between changes in their everyday life and the experience of building self-efficacy through doing activity in a safe and supervised space and becoming less fearful of being breathless:

They did explain during the course how, you know, if you got out of breath, to breathe through your nose, deep breath in and slow breath out and I found that good that helped. […] It wasn’t just the exercises it was… when they talk to you… that meant… to say that you could do the exercises. You’re not going to be…don’t worry about it, you’re not going to get out of breath. If you do get out of breath stop… and you sort of push yourself a little bit higher… I’m not out of breath, you know, I can do this […] Everyone said, I feel better doing [daily activities] (Participant 9).

## Discussion

The four dimensions of *doing, being, belonging* and *becoming* enable a way of understanding how the different aspects of this PR programme were experienced by participants. The narrative surrounding *doing* was most closely aligned to doing exercise and physical activity. Exercise is the cornerstone of PR (Spruit et al., 2013) and therefore the importance of exercise as a central feature of active engagement is not surprising. Although some participants reported health and functional benefits linked to feeling stronger, responses also suggested wider social and emotional benefits linked to doing exercises as part of a group, and gaining confidence from overcoming the perceived challenge of exercise. Such findings are consistent with wider literature (Desveaux et al., 2014).

However, our data suggests it is not simply exercise instruction that is important, but the embodied experience of doing exercise as part of the programme. The multi-layered parts of ‘doing’ – doing with others, doing with the supervision of a facilitator and coping when doing – all emphasised the importance of this experience. The experience of doing presented as contributing to feelings of self-efficacy. The idea of coping with challenging circumstances whilst ‘doing’ is also cited as a strategy for building self-efficacy with people with COPD (Selzlera et al., 2020).

Breathlessness is understood to be a distressing experience with widespread impact on quality of life (Hutchinson et al., 2018), and this was emphasised by the participants in this study. More specifically, the link between being breathless and doing physical activities within the programme had the potential to be both a facilitator to ‘becoming a person who lives with COPD’ and a difficult and uncomfortable experience. Participants discussed that the programme encouraged a ‘moderate’ degree of breathlessness during exercises in order to gain experience of this state of being and to gain confidence from managing and controlling this level. Some participants discussed a sense of success in experiencing breathlessness whilst doing activity, and being supported to use techniques in real-time in order to cope. However, it has been difficult to find consensus statements in literature about what constitutes moderate breathlessness, and it is therefore unsurprising that one participant voiced uncertainty about what this was and how to achieve it.

Adherence to PR is low with many patients either choosing not to take up the offer or not completing programmes (Oates et al., 2017). Studies have explored common barriers to adherence and patient characteristics which may predict poor adherence (Hayton et al., 2013; Oates et al., 2017). Our findings, particularly the application of the concept of belonging, add extra understanding about the initial thinking process when someone is considering attending PR. Examples of participants checking whether they were likely to belong (before the programme), or whether they could see themselves belonging (during early sessions) were frequently highlighted and related to both belonging within the social group and the physical space.

In all of these ways, influences on starting and continuing to engage in a PR programme is reflective of influences on engagement in occupations more broadly, with evidence suggesting that belonging to groups, belonging to stable places and even belonging to professionals or leaders underpins regular and sustained engagement in activity (Hitch et al., 2014). Such insights have important implications for those who refer to and deliver PR who should see their initial information and the initial experience of the first group as essential steps in creating motivation, purpose and the conditions for ongoing engagement. In simple terms, fitting in and feeling comfortable within the group, the venue and with facilitators are likely to be highly significant factors influencing whether people start or go back.

Features such as the importance of giving as well as receiving, and a respect for individual diversity in the midst of shared experiences (Hitch et al., 2014) should also raise important considerations for those striving to facilitate an optimum group environment. PR is typically delivered to groups (McCarthy et al., 2015) largely driven by the aim of facilitating activity and education simultaneously to optimum numbers of people. However, this study supports that belonging to a group holds far more power than simply reaching a group of similar individuals with the same standardised intervention.

We suggest that becoming a person who lived with COPD – with examples of increased confidence, more positive views of engagement in occupation and more positive visions of the future – was likely to be linked to a dynamic interaction of the other elements. A central belief of occupational therapists is that types of doing can lead to change and indeed doing and change become indivisible (Pentland et al., 2018). This was seen where participants reflected upon changes in terms of occupational engagement and things that they could now ‘do’. Change is perhaps most likely to be positive when people achieve something that is neither too easy or too hard, and construct meaning and purpose through doing (Pentland et al., 2018). Enjoyment of, and comfort within, sessions, a sense of belonging within both the social and physical environment and successfully using skills and strategies that had relevance in everyday life were all important here. It must be noted however that change may not always be positive, and this can be understood when thinking about the characteristics of doing. Examples of negative experiences of activities feeling unmanageable, or the strategy of moderate breathlessness being difficult to enact, affected the sense of meaning, purpose and ability to overcome challenge.

## Strengths and limitations

This is the only study, to our knowledge, which explores the service user experience of participating in an occupational therapy-led PR programme. Aligned with the goals of qualitative research, the small sample size enabled in-depth exploration of individual experiences. The use of multiple researchers, working within the framework analysis process, also added rigour to the process.

The change to telephone interviews ensured the research could still continue during COVID-19 movement restrictions and, whilst telephone interviewing for qualitative research is reported as well-received by participants, a potential limitation is the inability to read social cues which may support the researcher to add follow-up or clarifying questions (Ward et al., 2015).

Another point to note about the interviews taking place during the first national COVID-19 lockdown was that participant experiences were set against the context of issues such as limited access to services and facilities. As such, it is possible that participants were unable to take full advantage of the increased confidence they reported since, at the time, people were permitted to leave their homes only once per day. It is also possible that the participants who took part in this study were those who were most positive about the programme as it was more difficult to access those who did not take part or complete the programme.

## Conclusion

The findings of this study apply Wilcock’s Occupational Perspective of Health (2001a; 2001b, 2006) to propose a dynamic interaction between doing exercise, being (and coping with being) breathless and belonging as an individual within a group, and that these elements also contribute to becoming a person who lives with COPD.

Although exercise is reported as the central feature of PR, this study supports findings from wider literature (Brighton et al., 2020; Simony et al., 2021) that guiding people to, and during, physical activity within PR is a complex and multi-factorial challenge. Our study suggests that the embodied experience of ‘doing’ exercise, whilst experiencing ‘being’ breathless and a sense of ‘belonging’ as an individual to a group, were all factors in creating confidence, self-efficacy and a positive vision of ‘becoming’ a person who lives with COPD. We therefore propose that it is occupation that can be the central feature of PR and this framework can be applied as a framework for occupation-focussed PR programmes. This also leads to the recommendation that occupational therapists bring unique skills and knowledge to the facilitation of PR and can be instrumental in creating the conditions for positive engagement.

### Key findings

• ‘Doing, being, belonging and becoming’ provides an occupational perspective of the experience of participating in a PR programme, emphasising a way of understanding the programme as an occupation, and an experience which influences engagement in wider occupations.
•Those who plan and design PR could develop this as a framework for the delivery of occupation-focussed programmes.

### What the study has added

This study has added understanding of the service user experience of participating in an occupational-therapy led PR programme and this understanding can be used to design services which address its effectiveness.
